# Does the Composition of Gut Microbiota Affect Hypertension? Molecular Mechanisms Involved in Increasing Blood Pressure

**DOI:** 10.3390/ijms24021377

**Published:** 2023-01-10

**Authors:** Julita Tokarek, Emilian Budny, Maciej Saar, Joanna Kućmierz, Ewelina Młynarska, Jacek Rysz, Beata Franczyk

**Affiliations:** 1 Department of Nephrocardiology, Medical University of Lodz, ul. Zeromskiego 113, 90-549 Lodz, Poland; 2 Department of Nephrology, Hypertension and Family Medicine, Medical University of Lodz, ul. Zeromskiego 113, 90-549 Lodz, Poland

**Keywords:** gut microbiota, microbiome, hypertension, increased blood pressure, probiotics

## Abstract

Arterial hypertension is a chronic disease which is very prevalent contemporarily. The aim of this review was to investigate the impact of gut microbiota on the development and potential treatment of hypertension, taking into consideration underlying molecular mechanisms. The bacteria present in the intestines have the ability to secrete different metabolites, which might play a significant role in the regulation of blood pressure. The most important include short-chain fatty acids (SCFAs), vasoactive hormones, trimethylamine (TMA) and trimethylamine N-oxide (TMAO) and uremic toxins, such as indoxyl sulfate (IS) and p-cresyl sulfate (PCS). Their action in regulating blood pressure is mainly based on their pro- or anti-inflammatory function. The use of specifically formulated probiotics to modify the composition of gut microbiota might be a beneficial way of supportive treatment of hypertension; however, further research on this topic is needed to choose the species of bacteria that could induce the hypotensive pattern.

## 1. Introduction

Arterial hypertension (AH) is a chronic condition diagnosed when blood pressure (BP) value is elevated over 140/90 mmHg [1]. Arterial hypertension should be confirmed by at least two readings on two or more separate occasions [2].

Because of an aging population and increasing prevalence of hypertensive risk factors, such as overweight/obesity, dyslipidemia, physical inactivity, poor diet, low folate intake and excessive sodium intake, prevalence of AH increases with age: from 27% in patients aged younger than 60 years to 74% in those aged older than 80 years [2,3]. The World Health Organization estimates that 54% of strokes and 47% of ischemic heart disease are the complications of high blood pressure [4], which are responsible for 5.8% of all deaths worldwide [3].

Emerging evidence indicates that the gut microbiota is an important factor in the etiology of hypertension. Gut microbiota, a compound and highly varied community of microorganisms, live in the digestive tracts of humans and consists of several types of bacteria, archaea, eukaryote, viruses and parasites [5]. The gut microbiota of adult humans has been estimated to contain approximately 500–1000 distinct bacterial species; however, most of them belong to two of the major phylogenetic lineages: *Firmicutes* and *Bacteroidetes* [6]. The gut microbiota takes part in regulations of many metabolic processes, such as energy homeostasis and glucose and lipid metabolism [7]. Moreover, it performs basic functions in the immunological, structural and neurological landscapes of the human body [8]. Multiple studies revealed that AH is associated with lower gut microbiota diversity [9]. Higher salt intake, an important risk factor of AH, also affects gut microbiota composition, which equals an increase in *Lachnospiraceae*, *Ruminococcus* and *Parasutterella* spp. and decrease in *Lactobacillus* and *Oscillibacter* [10,11]. Moreover, dysbiosis could activate oxidation of LDL (low-density lipoprotein) to oxLDL which leads to vasoconstriction mediated by oxLDL and inhibition of nitric oxide (NO) synthesis—the most important vasodilator [12,13].

The aim of our study is to describe the role of gut microbiota in hypertension.

## 2. The Association between Gut Microbiota and the Development and Progression of Hypertension

The impact of gut microbiota on the human body has been analyzed by scientists for a long time.

Many scientific papers show that the gut microbiota could generate metabolic products, such as trimethylamine N-oxide (TMAO), that enlarge secretion of inflammatory factors, and hence, increase the risk of development of the atherosclerotic plaques [14,15]. Moreover, bacterial DNA has been found in these plaques [16].

Jing Li et al. specified two classes of enterotypes. The first enterotype was more opulent in *Prevotella*; on the other hand, the second one was more abundant in *Bacterioides*. The authors suggest that enterotype 1 of gut microbiota could be present in patients with prehypertension (pHTN) and hypertension (HTN), while enterotype 2 occurs in the healthy control group [17].

Scientists have proven that patients with high blood pressure present a higher capacity of bacteria, which should weaken gut barrier and cause intestinal inflammation [18]. Furthermore, animal studies show a reduction in the butyrate-producing bacteria in rats with hypertension [19].

Some bacteria might present higher abundance in hypertension. This correlation is presented in Table 1. Sun et al. suggested that there is a probability of higher quantity of *Anaerovorax*, *Butyricicoccus*, *Vampirovibrio*, *Methanobrevibacter*, *Oxalobacter*, *Cellulosibacter*, *Mogibacterium* and *Sporobacter* in hypertension [20]. Moreover, a higher capacity of *Alistipes finegoldii* and *Eubacterium siraeum* could be correlated with an increase of blood pressure [18,21]. Studies have reported that *Streptococcus* and *Parabacteroides*, *Salmonella* and *Eggerthella* could increase in the population with hypertension, in comparison to normal blood pressure [22]. Additionally, *Klebsiella* could have the same impact on blood pressure [17,22]. Furthermore, overgrowth of *Desulfovibrio* occurs in individuals with hypertension [17,21].

Researchers have shown that a population of particular bacteria could be present at a lower capacity in individuals with hypertension or higher in normal blood pressure. This correlation is presented in Table 2. J. Li et al. showed that the population of *Bifidobacterium*, *Coprococcus* and *Butyrivibrio* could have a higher occurrence in people with normal blood pressure [17]. Furthermore, a higher abundance of *Eubacterium rectale* was noticed in individuals with lower blood pressure [18,21]. Moreover, the populations of *Enterorhabdus*, *Romboutsia*, *Enterococcus*, *Lachnospiracea*, *Paraprevotella*, *Christensenella*, *Acetobacteroides* and *Coprobacter* appeared to be increased in healthy controls [21]. Additionally, *Roseburia* and *Faecalibacterium* might be present at lower amounts in hypertensive patients [17,22,23]. Furthermore, *Rothia*, *Atopobium*, *Anaeroglobus*, *Megaspheara* and *Pseudocitrobacter* might be present at lower quantities [20]. *Ruminococcus* also could exist in enriched amounts in patients with normal blood pressure [20,23].

A few bacteria present debatable changes in the healthy and hypertensive populations—included in Table 3. Jing Li et al. showed that *Oscillibacter*—a Gram-positive bacterium—could be enriched in a normotensive population; nevertheless, Shan Shun et al. exhibited that the amount of this bacterium should be decreased in comparison to people with hypertension [17,20]. The populations of *Prevotella* and *Clostridium* were described by X. Dan et al. as being decreased in individuals with hypertension; nevertheless, other authors have shown that the population of those bacteria should be elevated [17,21,22]. The probability of positive associations between hypertension and the genera *Bacteroides* was presented by X. Dan et al. [21]. Counter results of studies about *Bacteroides* were presented by S. Kim et al. [18].

The authors suggest that there is a need to undertake more detailed scientific research in order to define the association between the bacteria presented in Table 3 and hypertension. Researchers have indicated that studies considering division into specific bacterial species should be conducted.

## 3. Molecular Basis of Hypertension

Many different molecular mechanisms are involved in the process of maintaining arterial blood pressure. The most important of them are included in the following subsections.

### 3.1. Activation of the Renin-Angiotensin-Aldosterone System (RAAS)

**Figure 1:**
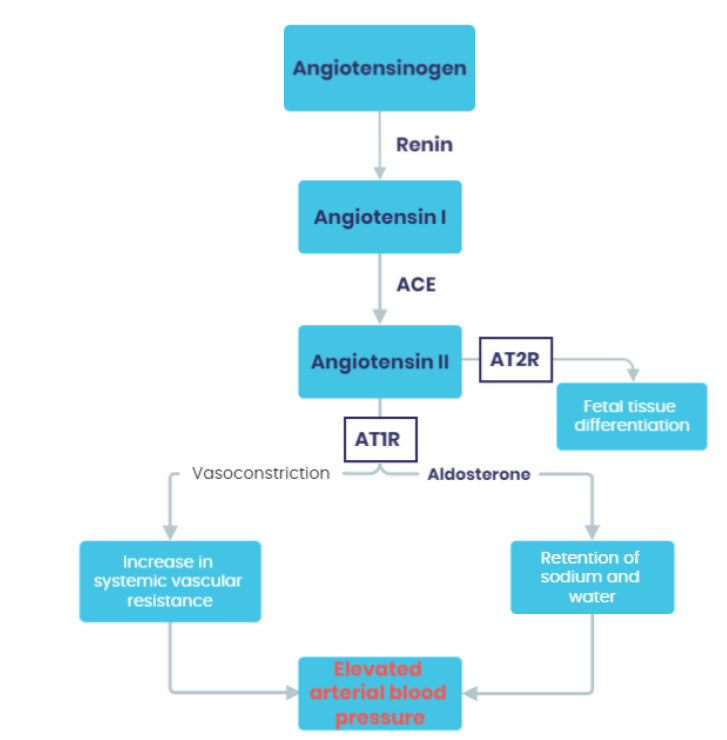
The scheme of the RAAS function. ACE—angiotensin converting enzyme; AT1R—angiotensin receptor type 1; AT2R—angiotensin receptors type 2.

Renin is a hormone produced in the juxtaglomerular epithelioid cells and released from granules in response to a decrease in renal perfusion pressure, hypovolemia or hyponatremia. Simultaneously, angiotensinogen is released from the liver in a continuous manner and is present in excess compared to renin. Renin acts as a proteolytic enzyme that converts angiotensinogen into angiotensin I (Ang I). Furthermore, angiotensin converting enzyme (ACE) secreted from endothelial cells converts angiotensin I to angiotensin II (Ang II). Ang II acts through angiotensin receptors type 1 (AT1R) and type 2 (AT2R). Stimulation of AT1R results in vasoconstriction, activation of sympathetic nervous system, aldosterone secretion and, hence, retention of sodium and water. These mechanisms lead to elevation of arterial blood pressure. On the other hand, stimulation of AT2R has the opposite effect on blood pressure, causing vasodilation and, additionally, anti-inflammatory and anti-fibrotic function. However, AT2R plays a vital role in the developing organism of fetus, but shows limited function in adults [24,25]. The scheme of the RAAS function is presented in Figure 1.

### 3.2. Baroreceptors

Arterial baroreceptors include carotid and extra-carotid baroreceptors. Carotid baroreceptors are located in the right and left carotid sinus and extra-carotid baroreceptors are found in the heart, in the aortic arch and in the pulmonary vessels [26]. These receptors have the ability to sense dynamic changes in the tension of the vascular walls and might regulate the activity of sympathetic nervous system (SNS) accordingly [24]. Although the significance of baroreflex mechanisms is mainly recognized in short-term regulation of blood pressure, its role in long-term maintenance of normal blood pressure remains the subject of studies. It is suggested that due to a persistent elevation of blood pressure, the baroreceptors might become less sensitive to conduct changes through SNS [27].

### 3.3. Adrenergic Receptors

The sympathetic nervous system (SNS) is of great importance in blood pressure regulation via the secretion of catecholamines that bind to adrenergic receptors (mostly α1 and β1). Stimulation of SNS results in an increase of the heart rate, total peripheral resistance and retention of sodium and water. Consequently, all these effects lead to the elevation of arterial blood pressure [28].

### 3.4. Natriuretic Peptides

The group of natriuretic peptides includes atrial natriuretic peptide (ANP), B-type natriuretic peptide (BNP) and C-type natriuretic peptide (CNP). These molecules originate in the myocardium and act as both endocrine and paracrine mediators. Activation of relevant natriuretic peptides receptors stimulates the synthesis of cyclic guanosine monophosphate (cGMP), which is responsible for the most vital effects of natriuretic peptides [29]. Therefore, ANP and BNP are deeply involved in the process of regulating blood pressure due to the diuretic, natriuretic and vasodilating effect demonstrated in response to increased pressure [30].

### 3.5. The Kinin–Kallikrein System (KKS)

The kinin–kallikrein system (KKS) is a complex multiprotein cascade that takes part in the activation of the intrinsic coagulation pathway and secretion of bradykinin-related peptides [31]. Bradykinin (BK) is a peptide involved not only in the inflammatory processes, but also the regulation of cardiovascular and renal function [31]. BK might increase the synthesis and secretion of nitric oxide (NO) and cGMP, and consequently, reduce the vascular tone and cause vasodilation. Furthermore, BK promotes diuresis and natriuresis, resulting in lower blood pressure. In order to ascertain the clinical relevance of KKS, studies have explored this topic and proved that reduced levels of renal kallikrein could be involved in the development of hypertension in both rodents and humans [32].

### 3.6. Microbiota-Derived Metabolites

This connection is described in detail in Section 4.

## 4. How Does the Microbiota-Derived Metabolites Interact with Hypertension at the Molecular Level?

**Figure 2:**
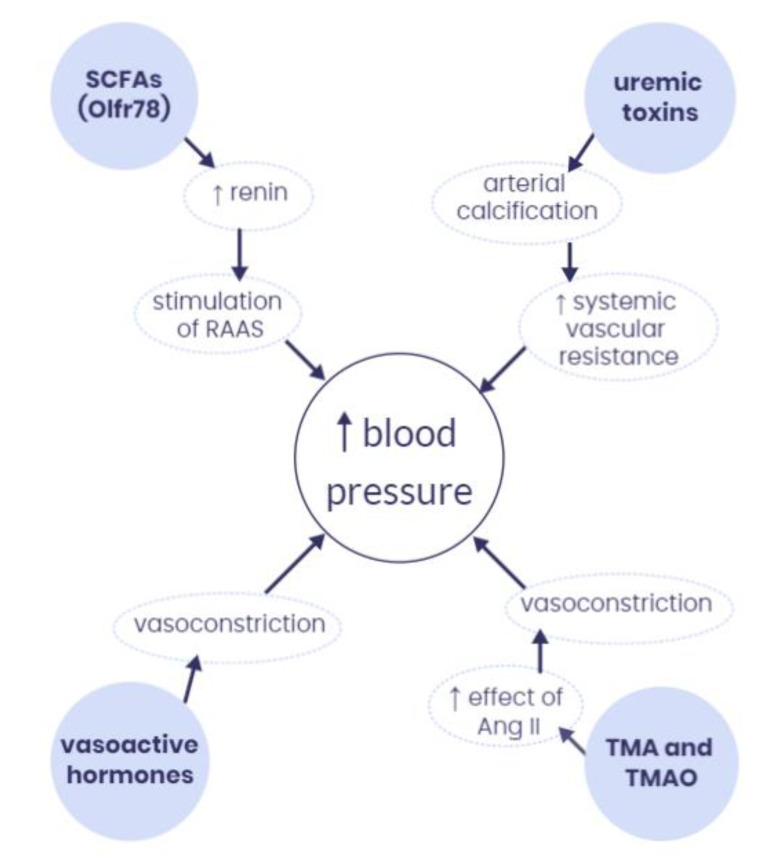
The most important microbiota-derived metabolites and their interactions with blood pressure. SCFAs—short-chain fatty acids; RAAS—renin-angiotensin-aldosterone system; Ang II—angiotensin II; TMA—trimethylamine; TMAO—trimethylamine N-oxide, Olfr78—olfactory receptor 78, ↑—increase.

The gut microbiota has the potential to produce a variety of metabolites that can reach the systemic circulation and act as signaling molecules that might regulate blood pressure in the host’s organism [33]. The most important microbiota-derived metabolites and their interactions with blood pressure are presented in Figure 2.

### 4.1. Short-Chain Fatty Acids (SCFAs)

Short-chain fatty acids (SCFAs) are fatty acids that have fewer than six carbon atoms, including mostly acetate (C2), propionate (C3) and butyrate (C4) [33]. They are the main product of bacterial fermentation of dietary fiber in the gut [24]. These molecules can be absorbed from the intestine via the gut epithelium, enter the circulating blood and bind to G-protein-coupled receptors (GPCRs), including GPR41, GPR43 and GPR109 (also known as FFAR3, FFAR2 and NIACR1, respectively), and GPCR olfactory receptor 51E2 (Olfr78 in mice and OR51E2 in humans), and therefore, regulate blood pressure (BP) [33,34].

Animal studies suggested that SCFAs can have both a hyper- and hypotensive effect, depending on the receptors involved. For example, SCFAs might take part in lowering BP thanks to the vasodilating effect of stimulation of GPR43 and GPR109a, which are present in renal arteries [9,35]. On the other hand, activation of Olfr78 in mice causes an increase in renin secretion from granules in the renal juxtaglomerular apparatus and stimulation of RAAS, which results in elevation of BP [36]. However, the hypertensive effect of SCFAs acting via stimulation of Olfr78, and its human analogue—OR51E2—was found to be weaker than the hypotensive effect of GPR43. These results suggested that Olfr78 might act as a negative feedback loop for GPR43 function [37].

Another mechanism in which SCFAs might regulate BP relates to the inhibition of histone deacetylase (HDAC) [38]. Animal models have shown that activated HDAC presents a proinflammatory effect and might be associated with a hypertensive phenotype in rats [39]. The anti-inflammatory effect of SCFAs on epithelial cells might be achieved via the inhibition of HDAC [9]. Especially butyrate (C4) is involved in this molecular pathway [40]. Moreover, butyrate modulates the secretion of many cytokines. It inhibits production of proinflammatory molecules, such as interleukin 12 (IL-12), tumor necrosis factor α (TNF-α) and interferon-γ (IFN-γ), and simultaneously, stimulates the production of anti-inflammatory particles, such as interleukin 10 (IL-10) [41]. Interestingly, the animal studies have even found that butyrate administration to rodents might alleviate inflammation and cause reduction of BP levels [42]. Butyrate producers that occur in greater amounts in normotensive phenotype include, e.g., *Roseburia*, *Rothia* and *Ruminococcaceaae* spp. [20,23].

Furthermore, SCFAs might also interact with central nervous system (CNS) via vagal receptors [9]. Animal studies have shown that higher levels of acetate (C2) in the large intestine might activate parasympathetic pathways and cause lowering of BP [43]. Consequently, this hypotensive effect of SCFAs appeared to be significantly inhibited in rats after vagotomy [43], which highlights the role of vagal stimulation in this mechanism.

### 4.2. Vasoactive Hormones

The studies have found that gut microbiota has the ability to produce vasoactive hormones, such as serotonin, dopamine and norepinephrine [44]. These molecules can be synthesized by several commensal bacterial genera, such as *Escherichia*, *Lactobacillus*, *Bifidobacterium* and *Streptococcus* [45]. All these neurotransmitters might be involved in the development of HA via the vasoconstrictive mechanism [46,47].

### 4.3. Trimethylamine (TMA) and Trimethylamine N-Oxide (TMAO)

Trimethylamine N-oxide (TMAO) is another gut microbiota-derived metabolite that can be involved in the compound process of BP regulation [48]. It originates from the microbial metabolism of trimethylammonium contained in, e.g., L-carnitine, choline and phosphatidylcholine. The first metabolite to be formed is trimethylamine (TMA), which undergoes further oxidation in the liver with the participation of flavin monooxygenases (FMOs), which convert TMA into TMAO [33]. Bacteria involved in carnitine and choline metabolism include among others: *Escherichia coli*, *Klebsiella* spp., *Citrobacter* spp., *Desulfovibrio alaskensis* and *Proteus mirabilis* [49].

Although the direct role of TMA and TMAO in the development of hypertension remains uncertain [50], studies have reported that TMAO might show the ability to prolong the hypertensive effect of angiotensin II [51,52]. It has been noticed in the literature that patients with chronic kidney disease (CKD) demonstrated higher plasma levels of TMAO [53,54]. The relationship between CKD and hypertension is considered bidirectional; therefore, elevated BP might be both the cause and effect of renal failure [55]. The recent animal-based study has found that rats receiving TMA solution presented increased arterial blood pressure (especially systolic). Moreover, rats administered a higher dose of TMA showed increased systemic vascular resistance, which might suggest the vasoconstrictive mechanism of observed phenomenon [50,56]. Although TMA and TMAO levels might not play a main role in the pathogenesis of arterial hypertension, they might contribute to the development and progression of the disease [57].

On the other hand, it is important to note that TMAO functions as a protective osmolyte against high osmotic pressure [58]. Furthermore, it has been reported that low-dose TMAO treatment in hypertensive rats might reduce diastolic dysfunction and heart fibrosis. This finding suggests that moderate increase of the plasma TMAO level could have a beneficial effect on the circulatory system [59].

### 4.4. Uremic Toxins

Microbiota-derived uremic toxins include among others indoxyl sulfate (IS) and p-cresyl sulfate (PCS) [33,60,61]. These molecules might cause arterial calcification due to proatherosclerotic, prothrombotic and proinflammatory effects [60,62]. Moreover, IS and PCS trigger oxidative stress, which also contributes to endothelial dysfunction [60]. All mentioned pathological changes to the arterial wall might lead to increased systemic vascular resistance, elevated BP and other cardiovascular complications.

Higher presence of bacteria correlated with a high IS level applies to *Bacteroides* and *Blautia*, while *Enterococcus*, *Akkermansia*, *Dialister* and *Ruminococcus* are associated with a higher PCS level [63].

## 5. Potential Use of Probiotics in Treatment of Hypertension

Hypertension is a common human condition, historically defined as a sustained elevation of systolic blood pressure (SBP) above 140 mmHg or diastolic blood pressure (DBP) above 90 mmHg. Essential hypertension (90% of patients) is a heterogeneous disorder, typically emerging in middle or elderly age, as a cumulative result of complex gene–gene and gene–environment interactions [64].

Luckily for the patients, there are numerous types of treatment available nowadays, mainly pharmacological, in which the leading drugs include diuretics, beta-blockers, angiotensin-converting enzyme inhibitors (ACE-I) and calcium channel blockers. Interestingly, minocycline, an antibiotic, presented a strong and positive impact on the regulation of blood pressure (BP) without harming the “healthy” microbiome [19] and to add importance, minocycline per os is able to increase the amount of cignuline, a protein that works as a tight junction in the intestines, thus preventing barrier disfunction [65] and decreasing the amount of inflammatory cytokines [66,67].

Naturally, diet is one possible way of dealing with some diseases, such as diabetes mellitus (DM), allergies or hypertension. Knowing that, it is vital to mention quercetin, which alters the microbiome through metabolites such as 3-(3-hydroxyphenyl)propionic acid, which reduces BP and works as a vasodilator [68,69,70].

Recently, the attention of researchers from all over the world has been focused on using probiotics to treat hypertension. The idea to use probiotics was based mostly on the fact that both bacteria and fungi might be beneficial in the long term, by inhabiting intestines and improving our health by secreting specific metabolites.

Our attention was focused on microbiota as well, because, e.g., enterochromaffin cells in mucosal and submucosal layer of stomach could be influenced by microbiota, as a consequence of serotonin, dopamine and norepinephrine release, which elevates BP because of changes that occur in the gut microbiome–brain axis. Recent studies discovered that in patients with hypertension (HT), dysbiosis occurs in certain bacterial families. Due to this fact, the term Firmicutes/Bifidobacterium ratio (F/B ratio) was created. It turns out that in patients who suffer from HT, that ratio is elevated; therefore, the amount of Firmicutes is larger than Bifidobacterium. Moreover, it is important to mention that the decrease of blood pressure might be a result of short-chain fatty acids (SCFA) production in bacterial cells [19,64,71,72].

Currently, studies cannot tell exactly what the proper genome of the microbiota is; however, it is assumed that even though there are differences at the phylum level, it is possible to observe some general directions in search of the “healthy” microbiome [73].

It was observed that implanting a diet enriched in *L. helveticus* strain was indeed able to lower blood pressure [74] and the more strains of bacteria, the more visible the result [75]. *L. plantarum* strain TENSIA has proven that this bacteria might be able to contribute to lowering BP in patients medicated with antihypertensive drugs, thanks to the increased excretion of water from the body [74]. Moreover, *L. plantarum* proved to have antimicrobial properties, and hence, it might play a protective role in maintenance of desirable “healthy” microbiome [76]. Interestingly, *L. animalis* might be able to create biologically active types of caseins that possess ACE-I functions [77].

Over time, many studies have been conducted, and some of them determined that in individuals with hypertension, the amount of Gram-negative bacteria could be elevated, especially of those belonging to the following genera: *Prevotella*, *Klebsiella*, *Parabacteroides* and *Desluvibrio* [17,18,21,23]. *Lactobacillus* is also able to help ameliorate hypertension by secreting substances working as Th-17 lymphocytes inhibitors that decrease inflammation; furthermore, it lower the BP [11] and might even be able to improve vascular function and insulin sensitivity, similar to *L. coryniformis* [78,79].

Even though still not enough studies considering the human microbiome have been conducted, it has been noticed that SCFA producers such as *Roseburia*, *Rothia* and *Ruminococcaceaae* spp. might be able to lower blood pressure [19,20,22,23,80]. Moreover, during treatment of hypertension with the use of candesartan, it has been observed that the amount of *Lactobacillus* could be increased in rodents [81], which may give us a chance to find the right pattern to treat hypertension. It was suggested that *Lactobacillus plantarum* has the ability not only to lower BP, but also decrease the level of fibrinogen, leptin (gut hormone stimulating hunger) and IL-6 (main proinflammatory interleukin) [82], and therefore, it might be able to reduce diastolic pressure within 8 weeks [83].

For starters, when it comes to treating many different diseases, the best way is to start low and go slow (if it is not a life-threatening state); therefore, dietary changes should be the first thing to alter, as well as psychological aspects such as behavior—e.g., avoiding stress and seeking psychiatric help if needed. Then, pharmacological therapy with the use of antihypertensives should be introduced, and, if all of the above options are currently involved, probiotics should be considered to alleviate symptoms of hypertension and augment the effects of methods that are already in use.

There is room to create a set of dietary rules (propositions of alimentation) for hypertensive patients that would become part of their life, and thus, it could make beneficial habits easier to follow for people regardless of their education, wealth or job.

Moreover, thanks to modern pharmacological technologies, we might be able to produce drugs that are precisely created to augment the proliferation and beneficial abilities of SCFA producing bacteria by:

○ Killing hostile microorganisms;
○ Developing drugs that work as growth inhibitors of unwanted bacteria species;
○ Creating substances that mimic function of SCFA so it could lower the price of probiotics and make it more accessible in regions where storing living organisms might be difficult (because of electricity deficiency or lower hygiene standards).

All the above-mentioned results of many studies suggest there might be a possibility to include probiotics into standard treatment of hypertension; however, further research on this topic is needed to fully establish the association between different bacterial species and regulation of blood pressure.

## 6. Conclusions

The association between the composition of gut microbiota and the development of arterial hypertension has been widely explored in the literature lately. The potential opportunity to alleviate the symptoms, and therefore, prevent further consequences of elevated blood pressure (BP) with the use of specific probiotics, has aroused great hopes.

The bacteria present in the intestines can produce and secrete different metabolites, which might play an important role in the regulation of blood pressure. The hypertensive effect is associated with increased level of microbiota-derived vasoactive hormones, trimethylamine (TMA) and trimethylamine N-oxide (TMAO) and uremic toxins, such as indoxyl sulfate (IS) and p-cresyl sulfate (PCS). The elevation of blood pressure might occur mostly due to the proinflammatory and vasoconstrictive function of these molecules.

Short-chain fatty acids (SCFAs) also belong to the group of bacterial metabolites involved in the process of blood pressure regulation. These particles might have both hypo- and hypertensive effect depending on the stimulated receptors, but their stronger effect applies to lowering the BP. Especially butyrate, produced by, e.g., *Roseburia*, *Rothia* and *Ruminococcaceaae* spp., contributes to the hypotensive effect via the anti-inflammatory action.

Moreover, it has been suggested that a greater population of different species of *Lactobacillus* (e.g., *L. helveticus*, *L. animalis*, *L. coryniformis* and *L. plantarum*) might be able to reduce the BP by promoting the anti-inflammatory phenotype.

Even though the dependence between the composition of gut microbiota and increased blood pressure has not been fully established yet, it is certain that patients with unbalanced microbial community are at a higher risk of developing hypertension. These conclusions might lead to potential new ways of supportive treatment of hypertension with the use of specifically formulated probiotics. The beneficial role in maintenance of normal BP attributed to *Lactobacillus* and *Bifidobacterium*, the most commonly found in probiotics, suggests that, until more detailed research is conducted, taking these probiotics might be considered as both primary prevention of hypertension and as an addition to standard therapies for elevated blood pressure.

This literature review has several limitations—it completely relies on previously published research, and even though the selection of the included data was done very meticulously, some errors resulting from previously applied methodology might occur. Moreover, the availably of non-open access research was limited due to lack of external funding.

The most important positive outcome of this scientific paper is that vital molecular mechanisms known for affecting the blood pressure in accordance with the composition of gut microbiota and their metabolites were gathered and discussed. Further studies on the species of bacteria which might be used in probiotics to achieve the significant hypotensive effect might be needed to implement this kind of therapy.

## Figures and Tables

**Table 1:** Presentation of the bacteria population representing a higher capacity in individuals with hypertension or lower capacity in people with normal blood pressure. The table contains the distribution for phylum, class, order, family, genus and gram classification.

Phylum	Class	Order	Family	Genus	Gram Classification	Presence in Hypertension
Actinomycetota	Coriobacteriia	Eggerthellales	Eggerthellaceae	*Eggerthella*	Positive	Higher [22]
Bacillota	Bacilli	Lactobacillales	Streptococcaceae	*Streptococcus*	Positive	Higher [22]
Bacillota	Clostridia	Clostridiales	Eubacteriaceae	*Anaerovorax*	Positive	Higher [20]
Bacillota	Clostridia	Eubacteriales	Eubacteriaceae	*Mogibacterium*	Positive	Higher [20]
Bacillota	Clostridia	Eubacteriales	Oscillospiraceae	*Butyricicoccus*	Positive	Higher [20]
Bacteroidetes	Bacteroidia	Bacteroidales	Porphyromonadaceae	*Parabacteroides*	Negative	Higher [22]
Bacteroidota	Bacteroidia	Bacteroidales	Rikenellaceae	*Alistipes*	Negative	Higher [18,21]
Euryarchaeota	Methanobacteria	Methanobacteriales	Methanobacteriaceae	*Methanobrevibacter*	Negative	Higher [20]
Firmicutes	Clostridia	Clostridiales	Ruminococcaceae	*Cellulosibacter*	Positive	Higher [20]
Firmicutes	Clostridia	Clostridiales	Ruminococcaceae	*Sporobacter*	Positive	Higher [20]
Proteobacteria	Deltaproteobacteria	Bdellovibrionales	Bdellovibrionaceae	*Vampirovibrio*	Negative	Higher [20]
Proteobacteria	Deltaproteobacteria	Desulfovibrionales	Desulfovibrionaceae	*Desulfovibrio*	Negative	Higher [17,21]
Pseudomonadota	Betaproteobacteria	Burkholderiales	Oxalobacteraceae	*Oxalobacter*	Negative	Higher [20]
Pseudomonadota	Gammaproteobacteria	Enterobacteriales	Enterobacteriaceae	*Klebsiella*	Negative	Higher [17,22]
Pseudomonadota	Gammaproteobacteria	Enterobacterales	Enterobacteriaceae	*Salmonella *	Negative	Higher [22]

**Table 2:** Presentation of the bacteria population representing a lower capacity in individuals with hypertension or higher capacity in people with normal blood pressure. The table contains the distribution for phylum, class, order, family, genus and gram classification.

Phylum	Class	Order	Family	Genus	Gram Classification	Presence in Hypertension
Actinobacteria	Coriobacteriia	Coriobacteriales	Coriobacteriaceae	*Enterorhabdus*	Positive	Lower [21]
Actinomycetota	Actinomycetia	Bifidobacteriales	Bifidobacteriaceae	*Bifidobacterium*	Positive	Lower [17]
Actinomycetota	Actinomycetia	Micrococcales	Micrococcaceae	*Rothia*	Positive	Lower [20]
Actinomycetota	Coriobacteriia	Coriobacteriales	Atopobiaceae	*Atopobium*	Positive	Lower [20]
Bacillota	Clostridia	Clostridiales	Eubacteriaceae	*Eubacterium*	Positive	Lower/Higher * [18,21]
Bacillota	Clostridia	Eubacteriales	Lachnospiraceae	*Butyrivibrio*	Positive	Lower [17]
Bacillota	Clostridia	Eubacteriales	Lachnospiraceae	*Coprococcus*	Positive	Lower [17]
Bacillota	Clostridia	Eubacteriales	Lachnospiraceae	*Roseburia*	Positive	Lower [17,22,23]
Bacillota	Clostridia	Eubacteriales	Oscillospiraceae	*Faecalibacterium*	Positive	Lower [17,22,23]
Bacillota	Clostridia	Eubacteriales	Peptostreptococcaceae	*Romboutsia*	Positive	Lower [21]
Bacillota	Negativicutes	Veillonellales	Veillonellaceae	*Anaeroglobus*	Negative	Lower [20]
Bacillota	Negativicutes	Veillonellales	Veillonellaceae	*Megaspheara*	Negative	Lower [20]
Bacteroidetes	Bacteroidia	Bacteroidales	Porphyromonadaceae	*Acetobacteroides*	Negative	Lower [21]
Bacteroidota	Bacteroidia	Bacteroidales	Porphyromonadaceae	*Coprobacter*	Negative	Lower [21]
Bacteroidetes	Bacteroidia	Bacteroidales	Prevotellaceae	*Paraprevotella*	Negative	Lower [21]
Firmicutes	Bacilli	Lactobacillales	Enterococcaceae	*Enterococcus*	Positive	Lower [21]
Firmicutes	Clostridia	Clostridiales	Christensenellaceae	*Christensenella*	Negative	Lower [21]
Firmicutes	Clostridia	Clostridiales	Lachnospiraceae	*Lachnospiracea*	Positive	Lower [21]
Firmicutes	Clostridia	Clostridiales	Ruminococcaceae	*Ruminococcus*	Positive	Lower [20,23]
Pseudomonadota	Gammaproteobacteria	Enterobacterales	Enterobacteriaceae	*Pseudocitrobacter*	Negative	Lower [20]

* Quantity of *Eubacterium* depended on species.

**Table 3:** List of phylum, class, order, family, genus and gram classifications in bacteria for which researchers have obtained different results in healthy and hypertensive populations.

Phylum	Class	Order	Family	Genus	Gram Classification	Presence in Hypertension
Firmicutes	Clostridia	Clostridiales	Ruminococcaceae	*Oscillibacter*	Positive	Higher [21]/Lower [17]
Bacteroidetes	Bacteroidia	Bacteroidales	Prevotellaceae	*Prevotella*	Negative	Higher [17,20,23]/Lower [20]
Bacteroidetes	Bacteroidia	Bacteroidales	Bacteroidaceae	*Bacteroides*	Negative	Higher [21]/Lower [18]
Firmicutes	Clostridia	Clostridiales	Clostridiaceae	*Clostridium*	Positive	Higher [17,20,23]/Lower [20]

## Data Availability

The data used in this article are sourced from materials mentioned in the References section.
